# Hybrid Open and Endovascular Repair in Pararenal Abdominal Aortic Pseudoaneurysm—Literature Review and Case Presentation

**DOI:** 10.3390/life15111765

**Published:** 2025-11-18

**Authors:** Vlad Anton Iliescu, Reza Nayyerani, Catalina Andreea Parasca, Pavel Platon, Catalin Baston, Bianca Morosanu, Ovidiu Stiru

**Affiliations:** 1 Faculty of Medicine, Carol Davila University of Medicine and Pharmacy, 050474 Bucharest, Romania; vlad.iliescu@umfcd.ro (V.A.I.); catalin.baston@umfcd.ro (C.B.); monica-bianca.morosanu@umfcd.ro (B.M.); ovidiu.stiru@umfcd.ro (O.S.); 2 Department of Cardiovascular Surgery, “Prof. Dr. C.C. Iliescu” Emergency Institute for Cardiovascular Diseases, 022328 Bucharest, Romania; 3 Department of Interventional Cardiology, “Prof. Dr. C.C. Iliescu” Emergency Institute for Cardiovascular Diseases, 022328 Bucharest, Romania; pavelplaton@yahoo.com; 4 Department of Urology, Fundeni Clinical Institute, 022328 Bucharest, Romania; 5 Department of Anesthesia and Intensive Care, “Prof. Dr. C.C. Iliescu” Emergency Institute for Cardiovascular Diseases, 022328 Bucharest, Romania

**Keywords:** pararenal abdominal aortic pseudoaneurysm, complex abdominal aortic aneurysm, hybrid treatment, ChEVAR, renal artery, mesenteric artery

## Abstract

Pararenal abdominal aortic aneurysm/pseudoaneurysms (PAAA/PAAP) are rare, high-risk complex aortic lesions involving the renal arteries. Management includes open surgical repair (OSR), endovascular aortic repair (EVAR), or hybrid repair, each with specific advantages and limitations. A review of the literature was performed to assess treatment strategies and outcomes for PAAA and PAAP. A PubMed search using relevant MeSH terms identified 184 articles published in the last five years. After applying inclusion and exclusion criteria, 34 studies comprising 6460 patients with complex AAA/AAP were included for analysis. Treatment strategies were predominantly endovascular (79.4%), followed by open (5.8%) and hybrid approaches (2.9%) (11.7% have used EVAR or OSR in the same study). To emphasize difficulties in the management of this pathology, a case report of a large PAAP involving both renal arteries and occluded celiac trunk with retrograde flow from patent superior mesenteric artery (SMA) is presented. Given the complex anatomy and high surgical risk, hybrid treatment was chosen consisting of bilateral ilio-renal Dacron bypasses followed by ChEVAR (chimney stenting of the SMA), with favorable postoperative recovery. The management of PAAP requires an individualized, anatomy- and risk-adapted approach. Open surgical repair remains preferable for younger, low-risk patients for superior long-term durability, whereas endovascular repair offers lower perioperative morbidity in high-risk cohorts. Optimal outcomes are dependent on high-volume centers with multidisciplinary expertise.

## 1. Introduction

Aortic pseudoaneurysms are defined as dilatation of the aorta due to disruption of all the aortic wall layers, being contained only by peri-aortic connective tissue [1]. Depending on the localization of the lesion on the reno-visceral segment of the abdominal aorta, the aneurysms or pseudoaneurysms are classified by the current European Society for Vascular Surgery guidelines as “complex abdominal aortic aneurysms (AAA)/pseudoaneurysm (AAP)” and represent about 15–20% of the all AAA. The pararenal subgroup presume the involvement of at least one renal artery (RA) but not the superior mesenteric artery (SMA) [1].

Owing to the anatomic relationship with the visceral branches, the repair of complex aortic lesions carries a higher risk (compared to that of infrarenal AAA) and demands careful evaluation and individualized treatment strategies. Treatment options for pararenal abdominal aortic pseudoaneurysms (PAAP) include open surgical, endovascular, or hybrid repair. Open surgical repair (OSR) involves suprarenal clamping, replacement of the affected aorta, and revascularization of the visceral arteries. On the other side, the endovascular repair became a novel alternative, being less invasive and having lower perioperative complication rates than OSR, especially in patients with significant comorbidities [2,3]. The continuous evolution of the endovascular technology offers, nowadays, several options including fenestrated endovascular aortic repair (FEVAR), branched endovascular aortic repair (BEVAR), parallel grafts (chimney EVAR (ChEVAR) or snorkel), and custom-made endografts, thus improving the results and extending the applicability of this technique [2,3,4,5]. However, there are some limitations to total endovascular repair of complex AAA, including unsuitable anatomy, excessive aortic angulations, inability to deliver the endograft, insufficient sealing zone, inability to reach the visceral vessels, complex customized devices, and procedural planning [4,6].

There are no randomized trials comparing the OSR with endovascular repair and only few studies try to address this, using the propensity score match [5]. The results are debatable in terms of the adequacy of comparison, considering the multiple differences between the study populations. Despite several adjustments in the perioperative care and surgical techniques, OSR is still associated with notable mortality rates [7]. Thus, open repair may be reserved for younger patients with low-surgical risk or unsuitable anatomy for endovascular treatment [3]. Meanwhile, the UK Complex Aneurysm Study (COMPASS) suggested a higher long-term mortality in the patients with endovascular treatment for complex AAA [2].

Therefore, hybrid open and endovascular repair of abdominal aortic lesions, consisting of a combined procedure between open reno-visceral revascularization and an endovascular approach, can be a viable treatment option in selected cases considered high-risk for OSR and inappropriate for EVAR [6].

Another aspect that must be highlighted is that the optimal treatment for complex abdominal aortic lesions is far from being established or standardized. It comprehends not only the operative time, consisting of multiple open, endovascular, or hybrid combinations, but it also includes adequate preoperative management (optimization and procedural planning) and the postoperative care (immediate ICU care, long-term follow-up, and medical rehabilitation) in order to achieve the best overall results.

## 2. Case Report

A 69-year-old male patient was admitted to our institute with a diagnosis of abdominal aortic aneurysm. The symptomatology consisted of diffuse abdominal pain starting 2 weeks before admission. The patient had multiple cardiovascular risk factors: systemic hypertension grade 3, dyslipidemia, smoking, age, and sex. His medical history included chronic pancreatitis, colonic diverticulosis, irritable bowel syndrome, and chronic obstructive pulmonary disease (COPD). At admission, the patient was in a good mental state, normoponderal (BMI 20 kg/m^2^), and was hemodynamically stable with a BP of 120/70 mmHg and a heart rate of 65 bpm. Physical examination revealed mild tenderness of the abdominal wall with pain in the peri-umbilical region and the left abdominal flank. No abdominal mass was detected on palpation and the patient did not have any gastrointestinal tract symptoms. Peripheral arterial pulses were detected in extremities in accordance with the heart rate. Laboratory findings revealed mild normochromic, normocytic anemia (Hb levels of 11.8 g/dL), nonspecific inflammatory syndrome (fibrinogen 697 mg/dL) and dyslipidemia (Cholesterol 169 mg/dl, LDL 120 mg/dL, HDL 42 mg/dL) with normal renal (Creatinine 1.02 mg/dL, Urea 12 mg/dL and eGFR 95mL/min) and hepatic functions.

**Figure 1:**
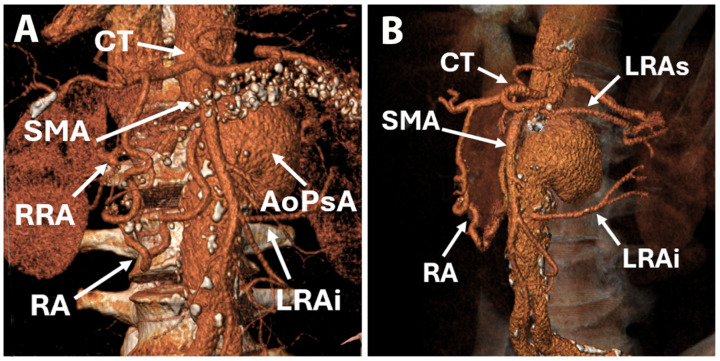
Computer tomography reconstruction of abdominal aorta and branches: anterior view (**A**), side view (**B**). AoPsA—aortic pseudoaneurysm; CT—celiak trunck; LRAi—left renal artery inferior branch; LRAs—left renal artery superior branch; RA—Riolan arcade; RRA—right renal artery; SMA—superior mesenteric artery.

As part of our preoperative investigation protocol, transthoracic echocardiography (TTE) revealed a normal left ventricle ejection fraction of 60%, no kinetic abnormalities, and mild aortic, mitral, and tricuspid regurgitations. Coronary angiography identified diffuse coronary artery disease without significant lesions: 30–40% stenosis of the left anterior descending artery (LAD), 60% stenosis of the first marginal branch (MG I), 60% stenosis of the right coronary artery (RCA). Abdominal computed tomography angiography (CTA) revealed perirenal and infrarenal abdominal aortic dilatation (diameter of 33 mm), with multiple atheromatous plaques and a pseudoaneurysm (67/63/64 mm) on the proximal left side of the abdominal aorta with peripheral circumferential thrombosis, without any contrast dye extravasation or active signs of bleeding (Figure 1).

**Figure 2:**
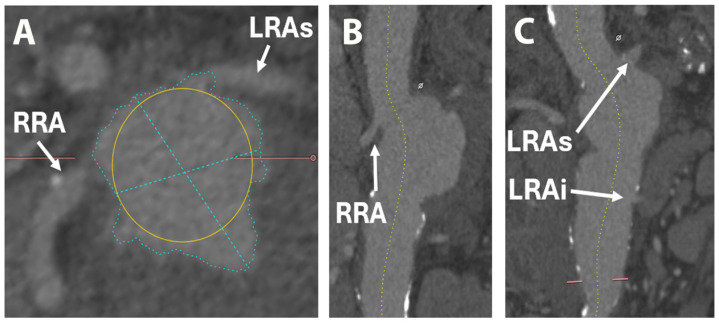
Computer tomography depicting origin of renal arteries: transverse view (**A**), modified coronal view (**B**,**C**). LRAi—left renal artery inferior branch; LRAs—left renal artery superior branch; RRA—right renal artery.

**Figure 3:**
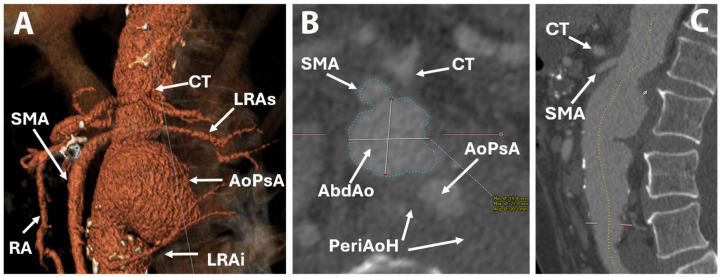
Computer tomography depicting origin of renal arteries: reconstruction (**A**), transverse view (**B**), and sagittal view (**C**). AoPsA—aortic pseudoaneurysm; CT—celiak trunck; LRAi—left renal artery inferior branch; LRAs—left renal artery superior branch; RA—Riolan arcade; SMA—superior mesenteric artery, AbdAo—Abdominal Aorta; PeriAoH—periaortic hematoma.

The right renal artery originated at the level of the pseudoaneurysm, and on the left side, there were two renal arteries with origins just cranial and caudal to the pseudoaneurysm (Figure 2). Thus, a diagnosis of PAAP was made. The proximal part of the celiac trunk was not visible, raising suspicion of ostial stenosis or thrombosis, despite the branches of the celiac trunk being visible. The SMA was dilated and permeable, with a diameter of 8 mm, starting 3 mm above the origin of the pseudoaneurysm (Figure 3).

**Figure 4:**
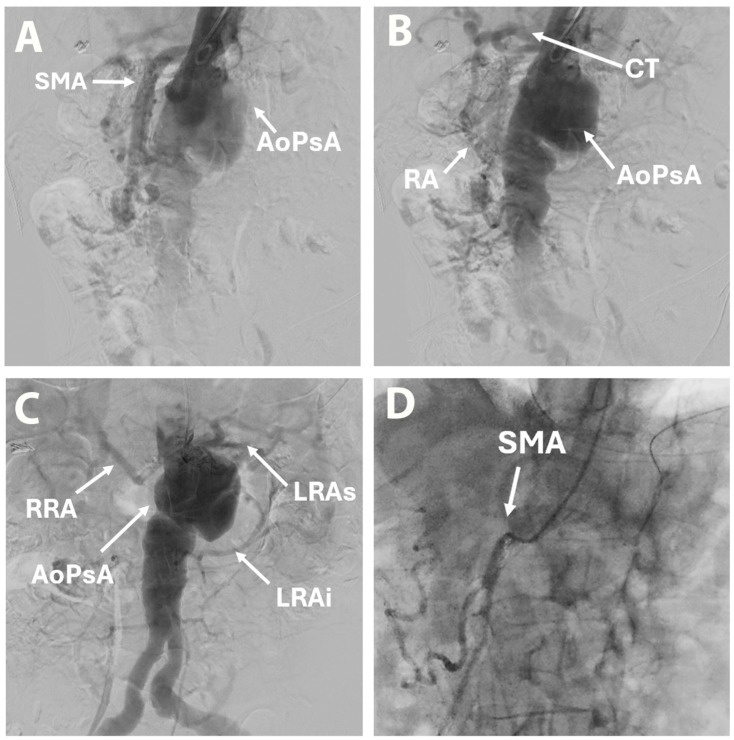
Aortography: initial view of mesenteric artery and aortic pseudoaneurysm (**A**), view of celiac trunk by retrograde flow from the superior mesenteric artery through the Riolan arcade (**B**), renal arteries distribution (**C**), selective arteriography of superior mesenteric artery (**D**). AoPsA—aortic pseudoaneurysm; CT—celiak trunck; LRAi—left renal artery inferior branch; LRAs—left renal artery superior branch; RA—Riolan arcade; RRA—right renal artery; SMA—superior mesenteric artery.

Besides some moderate atrophy and numerous calcifications suggestive of chronic pancreatitis, no other significant abnormalities were observed. Aortography revealed an AAP and permeable renal arteries, SMA, and celiac trunk. Selective arteriography of the celiac trunk was not possible, but retrograde flow from the superior mesenteric artery (SMA) through the Riolan arcade was confirmed (Figure 4). Angio CT analysis was performed using 3mensio Vascular 10.4 SP4 software, and treatment options were discussed by a multidisciplinary team. Considering the perioperative risk in the context of comorbidities and the particular anatomy, a decision was made in favor of a hybrid procedure with an open surgical renal artery bypass from the left external iliac artery, followed by ChEVAR with a single chimney to the SMA.

**Figure 5:**
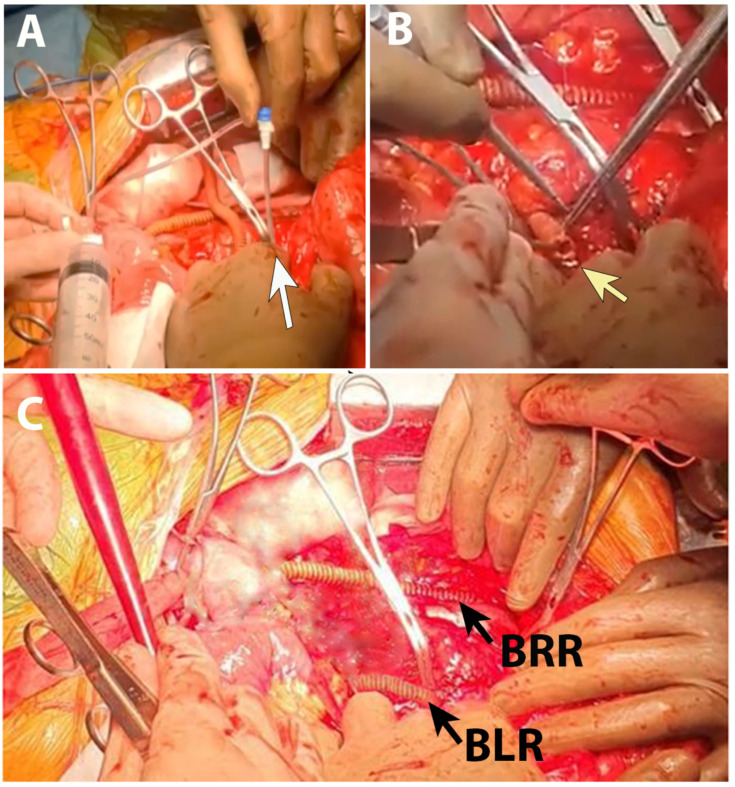
Intraoperative view: renoplegia administration in left renal artery inferior branch (**A**), termino-terminal anastomosis of graft to left renal artery inferior branch (**B**), and final aspect of renal revascularization (**C**). BRR—graft branch to right renal artery; BLR—graft branch to left renal artery inferior branch.

**Figure 6:**
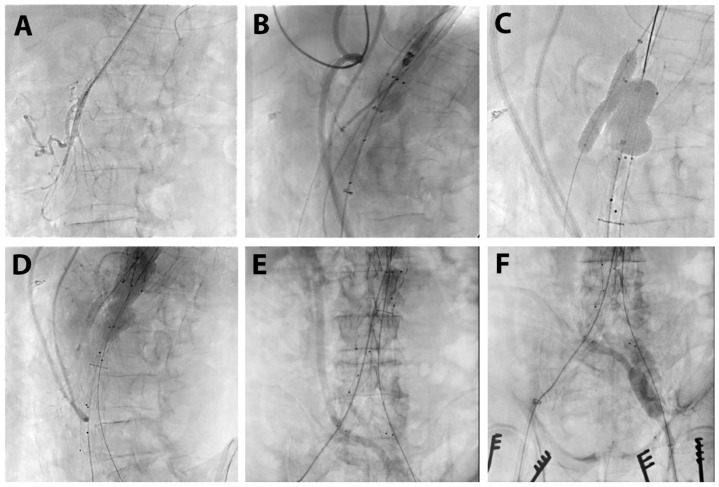
Intraprocedural angiographic aspect: selective cannulation of SMA and stent introduction (**A**), endoprosthesis positioning above SMA origin (**B**), concomitant postdilatation of proximal endoprosthesis and SMA stent (**C**), angiographic view of proximal aorta with chimney on SMA (**D**), angiographic aspect bifurcated graft to renal arteries (**E**,**F**).

Preoperative preparation included standard and invasive monitoring using a left radial artery catheter, a central venous line in the right internal jugular vein, and urinary catheterization. A peridural catheter was inserted at the L2–L3 level for postoperative pain management. Antibioprophylaxis was ensured with Cefuroxime and Gentamicin. The procedure was performed under general anesthesia with orotracheal intubation. The surgical procedure started with a median laparotomy, entry into the peritoneal cavity, and forward, with a right transperitoneal approach, exposing the abdominal aorta and inferior vena cava. The right renal artery was identified and isolated. On the left side, the inferior renal artery was identified and isolated, but the superior renal artery was only identified surrounded by the perianeurysmal hematoma. This was followed by identification and isolation of the left external iliac artery, which was clamped proximal and distal to the anastomosis site, and a 14/7/7 bifurcated Dacron prosthesis was sewn in a termino-lateral fashion. The right renal artery was clamped, a small arteriotomy was performed, and cold renoplegia (350 mL Custodiol solution) was administered. Total arteriotomy and termino-terminal anastomosis between the right renal artery and 7 mm Dacron branch were performed. The same sequence was performed on the left side with the left inferior renal artera (Figure 5). Bilateral Scarpa trigone incisions were made, and the right and left femoral arteries were identified and isolated. A no. 9 BeGraft (Bentley Innomed, Hechingen, Germany) was introduced via the right brachial artery and positioned in the SMA. The main body of the endoprosthesis (MEDTRONIC ENDURANT IIs 32/16/166) was inserted under fluoroscopic guidance from the right femoral artery using a 20Fr SENTRANT sheath and positioned 1.5 cm above the origin of the SMA. The proximal part of the endoprosthesis was dilated with a balloon concomitant with the expansion of the SMA stent. A left common iliac artery extension (MEDTRONIC ENDURANT 16/16/156) was inserted from the left femoral artery, followed by a right iliac artery extension (MEDTRONIC ENDURANT 16/16/82) via the right femoral artery. Postdilatation with balloon at the endoprosthesis junctions, aortic bifurcation, and iliac arteries was made. Intra-procedural control aortography showed an adequate position of the endoprosthesis without any endoleaks, a permeable SMA, celiac trunk with retrograde flow, and permeable renal bypasses (Figure 6).

The intraoperative blood loss was 300 mL and we used the Cell SaverElite autotransfusion system (Haemonetics Corp., Boston, MA, USA). No blood products were administered intraoperatively and the maximal dose of vasopressor support was 190 ng/kgc/min of Noradrenaline.

**Figure 7:**
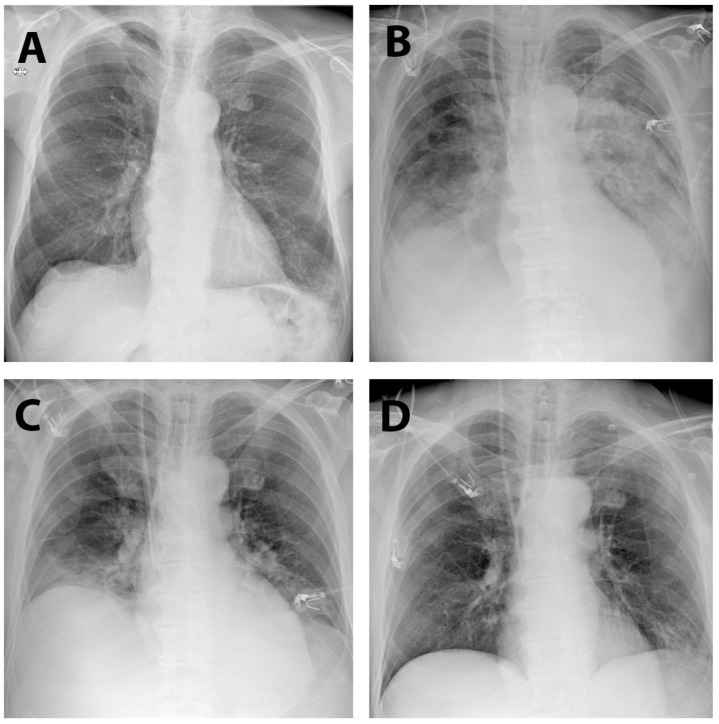
Chest X-ray series: preoperative aspect (**A**), during acute respiratory failure (**B**), improvement in lung infiltrates (**C**), and at discharge (**D**).

**Figure 8:**
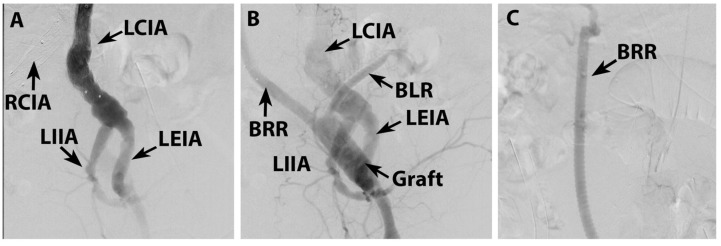
Postoperative control angiography: early visualization of distal part of endoprosthesis and patent iliac artery and branches (**A**), late visualization of iliac artery and branches, with patent ilio-birenal graft (**B**), and patent graft to right renal artery and branches (**C**). BLR—graft branch to left renal artery; BRR—graft branch to right renal artery; LIIA—left internal iliac artery, LEIA—left external iliac artery; LCIA—left common iliac artery; RCIA—right common iliac artery.

The initial postoperative evolution was favorable, and the patient was extubated 12 h after ICU admission, with mild–moderate doses of noradrenaline (100 ng/kgc/min), maximal lactate level of 1.2 mmol/L, 1500 mL diuresis, and no neurological dysfunction. However, on postoperative day 2, the patient developed acute respiratory insufficiency requiring orotracheal intubation. The patient tested positive for influenza type A, and treatment with oseltamivir was initiated (Figure 7). This was followed by systemic inflammatory response syndrome, which progressed to septic shock with pulmonary origin, requiring moderate doses of vasopressor support. As the patient developed oligoanuria with acute kidney injury (increase in serum creatinine up to 4.7 mg/dL and urea up to 125 mg/dL), hemodialysis was initiated. Renal infarction with possible additional contrast dye nephrotoxicity was suspected; therefore, we performed control arteriography. This revealed permeable ilio-renal bypasses and SMA and celiac trunk branches, with no signs of endoleak. (Figure 8). However, increasing lactate levels raised the suspicion of mesenteric ischemia, which was excluded after exploratory laparotomy. The patient underwent dialysis with CytoSorb for 7 days, during which progressive improvement in renal function was observed, marked by the normalization of creatinine, urea, and lactate levels, recovery of diuresis, and remission of inflammatory markers. Antibiotic prophylaxis was also administered, especially in the context of pre-existing COPD, and the patient’s condition improved. He was extubated on postoperative day 10, and the ICU stay was 14 days; he was discharged on postoperative day 17. He returned at 1 month follow-up with good clinical status, and abdominal echography was normal.

Written informed consent was obtained from the patient for the publication of the case, medical data, and images. The study was approved by the Institutional Ethics Committee.

## 3. Discussions and Review of the Literature

Aortic pseudoaneurysms, also called “false aneurysms”, are defined as dilatation of the aorta due to disruption of all the aortic wall layers, being contained only by peri-aortic connective tissue, and they may be a result of the progression of a penetrating aortic ulcer (PAU) [1]. Usually, these types of lesions are found in elderly patients, mostly caused by systemic atherosclerosis, which was also the case for our patient. The incidence is estimated around 1% in the vascular population, and the abdominal localization (11–24%) is rarer than the thoracic one (76–86%) [1]. Up to 70% of the cases are symptomatic, with abdominal pain (back or flank pain) being the most frequent (52%), followed by acute lower limb ischemia due to distal embolization (12%) or rupture (7%), thus giving the urgent nature of the treatment. The guidelines highlight the notion of “complicated PAU” referring to the presence of the pseudoaneurysms, embolic episodes, and recurrent pain. Our patient did not manifest or describe any lower limb embolisms, but recurrent abdominal pain and the pseudoaneurysm were present.

Abdominal aortic aneurysms that extend into the reno-visceral segment of the abdominal aorta, but without involvement of the thoracic aorta, are collectively categorized as complex AAAs. According to the current European Society for Vascular Surgery (ESVS) guidelines, this group encompasses several distinct anatomical subtypes, classified primarily by the relationship of the aneurysmal disease to the visceral and renal branches. These include the following: short-neck infrarenal AAAs, defined by an infrarenal neck length between 4 and 10 mm; juxtarenal AAAs, characterized by an infrarenal neck shorter than 4 mm, yet without direct involvement of the renal arteries; pararenal AAAs, which involve at least one renal artery but spare the superior mesenteric artery; paravisceral AAAs, involving both the renal arteries and the SMA, but not the coeliac trunk; and suprarenal AAAs, a designation that often groups together pararenal and paravisceral aneurysms due to their anatomical and therapeutic similarities. The most extensive form within this classification is the type IV thoracoabdominal aortic aneurysm (TAAA IV), which affects the renal arteries, SMA, and celiac trunk, thereby encompassing the entire abdominal aorta from the level of the diaphragm to the aortic bifurcation [1]. Importantly, this anatomical classification is equally applicable to pseudoaneurysms arising in the same segment, given their comparable location and branch involvement. In our case, the CTA revealed a PAAP with diameters of 67/63/64 mm with peripheric circumferential thrombosis, located on the proximal left side of the abdominal aorta. However, in some patients, anatomical particularities represent an interesting point on this classification. The distribution of the reno-visceral branches is not always the same. They may be distributed on a longer distance on the aorta, having more space between them, or sometimes they may be very close to each other. Here, the SMA was not involved but the origin was just 3 mm above the origin of the pseudoaneurysm. In particular cases, the number of reno-visceral branches may be different, as secondary branches may have direct origin from the aorta or additional renal artery could be present, like in our case where the right renal artery had the origin in front of the pseudoaneurysm, but on the left side there were two renal arteries with origin just cranial and caudal to the pseudoaneurysm.

The diagnosis of PAU or complicated PAU with pseudoaneurysm is put on imagistic evaluation as both CTA and magnetic resonance angiography (MRA) have a high grade of accuracy. While both are useful, only the CTA can be used for preoperative planning, giving accurate measurements and establishing the opportunity for an endovascular treatment.

The current European Society for Vascular Surgery (ESVS) guidelines recommend, as Class IIa, that patients with AAP or PAU should be considered for surgical treatment, preferably endovascular. The same guideline recommends a more aggressive approach for an invasive treatment in complicated PAU, intramural hematoma, or isolated abdominal aortic dissection associated with concomitant aortic diameter over 30 mm [1]. This was also applicable in our case, where the CT scan showed, in addition to the PAAP, an infrarenal abdominal aortic dilatation with a diameter of 33 mm.

The close anatomical relationship between complex aortic lesions and the visceral branches makes their repair considerably more challenging than that of standard infrarenal AAA, necessitating thorough preoperative assessment and highly individualized treatment strategies. Management options for PAAPs include open surgical, endovascular, and hybrid approaches.

Open surgical repair typically requires suprarenal clamping, replacement of the diseased aortic segment, and reimplantation or bypass of visceral arteries. The placement of the proximal aortic clamp represents a critical and technically demanding aspect of the procedure. Compared with inter- or infrarenal clamping, supraceliac clamping has been consistently associated with higher perioperative mortality, increased risk of postoperative renal dysfunction, and a greater incidence of unplanned reoperations [8]. Multiple studies reported in the literature have demonstrated that OSR performed by high-volume surgeons is associated with reduced mortality and complication rates [8,9]. An important determinant of outcome that should not be overlooked is the influence of institutional case volume. Evidence from registry data indicates that high-volume centers, defined as those performing more than 14 open JAAA repairs annually, achieved significantly lower adjusted perioperative mortality (3.9%) than low-volume centers. These findings suggest that the broader implementation of centralization strategies for the management of JAAA and PAAA could further enhance patient outcomes, particularly in the context of OSR [8,10].

Although OSR remains a durable option, endovascular repair has become an increasingly attractive alternative, particularly in patients with significant comorbidities, due to its minimally invasive nature and lower perioperative morbidity and complication rates [2,3]. Advances in endovascular technology have substantially expanded the therapeutic spectrum, offering several options such as FEVAR, BEVAR, parallel graft techniques such as ChEVAR or snorkel configurations, and custom-made endografts. These developments have improved clinical outcomes and broadened the applicability of endovascular repair in anatomically complex scenarios [2,3,4,5]. Nevertheless, several limitations persist, including unfavorable anatomical features, excessive aortic angulation, insufficient sealing zones, difficulties with device delivery, and the inherent complexity of customized endografts [4,6].

While FEVAR demonstrates comparable postoperative mortality rates to OSR and appears to be superior with regard to perioperative morbidity, it is associated with a higher incidence of secondary interventions and its applicability is often limited by anatomical constraints [8]. A relevant contributor to this increased reintervention rate may be persistent type II endoleaks, reported in several studies within the FEVAR cohorts, although frequently without a detailed specification of the underlying mechanisms. Moreover, contemporary FEVAR for JAAA repair typically involves four-vessel fenestrations, which, by extending the proximal extent of repair, may predispose patients to additional mid-term complication [11]. The necessity for repeated reinterventions, coupled with the substantial cost of advanced endovascular devices, raises concerns regarding the broader adoption of this technique. In a study by Michel M. et al., FEVAR was found to be more expensive and not cost-effective for JAAA and PAAA within a two-year follow-up [10,12].

Another endovascular solution that proved its advantages in the treatment of JAAA and PAAA is ChEVAR, particularly in emergencies or in patients with unsuitable anatomy for FEVAR. However, major concerns were raised when using more than two chimneys, regarding the incidence of type IA endoleak and the durability of the technique, due to the gutters created between the chimneys and the main body of the endograft [10,13].

In our case, we performed an extended preoperative evaluation and discussion with a multidisciplinary team composed of cardiovascular surgeons, vascular surgeons, intensivists, anesthesiologists, cardiologists, and radiologists. We considered all surgical and endovascular options. Open surgical repair would have to consider a high-origin lesion located 3 mm from the superior mesenteric artery (SMA), with the celiac trunk occluded at its origin. Visceral perfusion was dependent on retrograde flow from the SMA via the Arch of Riolan. Consequently, all visceral branches were reliant on the SMA, necessitating reimplantation or bypass during open repair, thereby significantly increasing the procedural complexity. Another critical consideration was the presence of a pseudoaneurysm, which, according to current guidelines, warrants preferential management via an endovascular approach. FEVAR was not available at our institution; however, given the anatomy in our case and considering the localization of the PAAP and the three renal arteries, the risk of endoleak was very high. Total ChEVAR was also not feasible because of the small aortic diameter of the neck (24 mm) in that area, which did not allow the two additional chimneys and the main endoprosthesis. Considering the anatomical particularities of this case, we decided to perform a hybrid approach with ChEVAR, a single chimney to the SMA, and open surgical renal revascularization with retrograde iliac-renal bypass using a bifurcated Dacron prosthesis. By using this approach, we avoided the possible complications of a very demanding surgical repair with revascularization of the renal and visceral arteries and simultaneously overcame the limitations of the endovascular treatment.

Numerous studies have assessed the durability of retrograde bypasses, addressing a key concern in hybrid endovascular and surgical repair. In a retrospective review conducted by Kansal et al., there was no difference between antegrade and retrograde bypasses in long-term durability [14]. Another study by Quinones-Baldrich et al. reported no complication related to the retrograde grafts for visceral revascularization [3]. Moulakakis et al. reported excellent patency in the revascularized vessels (the pooled estimate for visceral graft patency rate was 96.5% (95% CI, 95.2–97.8%)) at a mean follow-up of 34.5 months [7].

In terms of perioperative complications, defined as complications within 30 days, the literature divides them into major complications and vascular complications based on Clavien–Dindo classification [15]. The major complications are represented by the following: intraoperative blood loss over 5000 mL, reintervention for bleeding, bowel ischemia, myocardial infarction, stroke, spinal cord ischemia or paraparesis, prolonged ICU stay, and renal disfunction with need for renal replacement therapy or multi-organ failure. The vascular complications include the following: iatrogenic vessels damage, occlusion of the revascularized branch, occlusion of the graft, graft infection, distal embolization, limb ischemia, and major amputation [2,16]. A study conducted by Yu et al. comparing the outcomes after OSR vs. FEVAR for JAAA using data from the Swedvasc Registry found that the incidence of perioperative major complications was higher in the OSR group (19.3%) than in the FEVAR group (7.4%) with significantly higher rates of prolonged ICU stay, bowel ischemia, and intraoperative blood loss. In the same study, the incidence of vascular complications was slightly higher in the FEVAR group (11.9%) than OSR group (8.8%) with iatrogenic vessel injury being the most frequent complication. Renal replacement therapy was required in 5% of the patients included in the study [2].

Zlatanovic et al. published some results from the Juxta- and Pararenal Aortic Aneurysm Multicentre European Study (JAMES) comparing the OSR and endovascular repair. Total blood loss, number of patients requiring blood transfusion, and total number of transfusions were higher in the OSR group, which was an expected result. In the OSR group, the ICU stay was longer than the endovascular group, as well as acute kidney injury being more frequent after open surgical repair (40.7% vs. 24.8%). In OSR, prolonged clamping time causing post-ischemic tubular necrosis can explain acute kidney injury. Other causes may be technical issues, embolic events, or contrast-induced nephrotoxicity [5]. The incidence of renal failure after OSR, is reported to be between 12% and 39% [17]. In our case, the patient experienced acute renal injury with oligoanuria and increasing renal function markers, requiring hemodialysis. Several factors contributed to this. On one hand, it was a systemic inflammatory response syndrome (SIRS) triggered by the septic shock requiring moderate doses of vasopressor support. Additionally, the patient suffered a renal infarction. Despite attempts to revascularize renal arteries, the anatomical peculiarity of having two left renal arteries was a critical factor in this case. The left superior renal artery was encased by the perianeurysmal hematoma, making revascularization impossible. It became evident that revascularizing only one of the left renal arteries was insufficient in our case. Another important aspect is the administration of renoplegia during surgery. In our case, we administered 350 mL of cold Custodiol solution to protect the kidney; however, there is no standard approach described in the literature regarding the indication of administration and the duration of ischemia. Further studies are required to address this issue. Additionally, contrast-induced nephrotoxicity should be considered. Usually, in a patient with normal renal function, it does not have such a great impact, but in a patient with a possible renal infarction, SIRS, multiple organ dysfunction syndrome (MODS), and systemic vasoconstriction, it could only worsen acute renal insufficiency. The question that should be evaluated in the future is when the best moment is to start hemodialysis, considering the amount of contrast dye administered in the preoperative phase (for CTA or aortography) and intraoperatively.

One of the most common complications after endovascular treatment is the endoleak. CTA is the most sensitive investigation to identify the endoleaks [18]. Zlatanovic et al. reported a higher rate of aortic-related reinterventions in the endovascular group, with the most frequent cause being type II endoleak [5]. Moulakakis et al. reported that 22.7% of the patients presented with endoleak during the follow-up (28.5% type I, 60.5% type II, 10.9% type III) and in 22.7% of these cases, a reintervention was needed [7]. Yu et al. also reported six type III and only two type I endoleaks in the cases treated with FEVAR [2]. For the patients treated with the hybrid approach, at 16.6 months mean follow-up, 10.5% required reintervention for type I and type II endoleaks [3]. Type I endoleak is particularly associated with accelerated sac dilatation and an increased risk of aneurysm rupture [5].

Owing to the anatomical localization of complex AAA and its relationship with the visceral arteries, treatment, either by OSR or endovascular, poses some challenges regarding mesenteric perfusion. Bowel ischemia, bowel resection, reintervention, and prolonged ICU stay are all possible consequences of mesenteric malperfusion. As Moulakakis et al. reported, the pooled estimate for mesenteric malperfusion in the selected studies was 5.2% [7]. In our case, although it was a PAAP, the superior margin was very close to the SMA. Concomitantly, the celiac trunk was occluded proximally. Thus, all the visceral vascularization was supplied by the SMA, making it one of the key points for a successful result. We decided to perform a ChEVAR with a single chimney to the SMA because the aortic diameter in that area did not support more than one chimney and the main body of the endoprosthesis. Studies have reported safe and satisfactory results for the use of stent on SMA even in acute aortic dissection complicated with mesenteric malperfusion [19]. In our case, given the early postoperative evolution, mesenteric ischemia was suspected despite technically satisfactory intraoperative results. We performed exploratory laparotomy, which revealed normal appearance of the intestines and other abdominal organs. However, antibiotic prophylaxis was administered in case of “gut-leak” syndrome.

Age has been consistently identified in the literature as a significant risk factor for postoperative complications and increased mortality, with some studies indicating up to a five-fold higher 30-day mortality among patients over 80 years old undergoing FEVAR for complex AAAs [2]. However, this was not the case for our patient.

The treatment strategy does not end with patient discharge. It comprehends the immediate postoperative and long-term follow-up, as well as medical recovery through a rehabilitation program. A complete treatment should provide the patient with a good quality of life (evaluated by different parameters) and social reintegration. CTA, laboratory tests and clinical examination are all part of a follow-up protocol which is recommended to be performed at 1, 3, 6 months and then annually [6].

We reviewed the literature to identify the treatment strategy and reported 30-day mortality and long-term survival rates for PAAA and PAAP. We searched the PubMed database using the syntax (pararenal abdominal aortic aneurysm) OR (pararenal abdominal aortic pseudoaneurysm) with the afferent MeSH terms (abdomen; aorta; aortic aneurysm, abdominal; aortic diseases; aortic aneurysm; aortic aneurysm, thoracoabdoninal; aortic aneurysm, familial abdominal; aneurysm, false). The advanced search returned 184 manuscripts published in the last five years. The inclusion criteria were as follows: manuscripts published in the last 5 years in trusted journals, manuscripts that included treatment strategies and short- or long-term follow-up, and studies that reported mortality or survival rates. The exclusion criteria were as follows: duplicate manuscripts, manuscripts that did not have access to the full version, manuscripts in a language other than English, Manuscripts that presented only the ethiology or pathogenesis of the disease, manuscripts without a description of the treatment, studies not including pararenal aneurysms or pseudoaneurysms, systematic reviews or case presentations, experimental studies on animals, and comments on published articles. Two independent reviewers screened titles and abstracts, followed by full-text assessment. Discrepancies were resolved by consensus with a third reviewer. After applying the inclusion and exclusion criteria, 34 articles were selected for review. For each selected study, we extracted the following: number of patients, average age, aneurysm type, treatment strategy, 30-day mortality, survival rates, and mean follow-up duration, if available. Data were entered into a structured table for comparative analysis (Table 1).

In total, 6460 patients treated for complex AAA/AAP were included. The endovascular treatment strategy was used in 79.4% of the selected studies, while 11.7% included endovascular or OSR, 5.8% open surgical, and 2.9% hybrid treatment. The results are presented in Table 1.

In our case, the 30-day mortality was 0%, which is consistent with previously published data showing 30-day mortality rates between 0% and 3% for hybrid or endovascular procedures in PAAP reported by Reyes et al. and Branzan et al. [21,31]. For the hybrid treatment strategy, Escobar et al. reported a 14.4% 30-day mortality [4].

## 4. Limitations

This case report offers insight into hybrid repair of PAAP within the broader context of treatment options through a supporting literature narrative review, though several limitations must be acknowledged. While the literature review included 34 studies, the absence of randomized controlled trials (RCTs) comparing open, endovascular, and hybrid approaches limits the strength of evidence, with most data coming from observational studies susceptible to bias. The review may also be affected by selection and publication bias, as it included only English-language, PubMed-indexed articles from the past five years. By being a single-case report, generalizability is not possible to broader patient populations. Additionally, the case’s short-term follow-up does not allow conclusions about medium- and long-term outcomes or complications. Another limitation is the influence of institutional device availability, as the unavailability of FEVAR technology led to the choice of hybrid repair, which may not apply in centers with broader resources. Finally, aspects of intraoperative care, such as renal protection strategies and timing of dialysis, are not standardized, leaving room for clinical uncertainty. These limitations highlight the need for multicenter studies and long-term data to better guide treatment strategies for PAAP.

## 5. Conclusions

The management of PAAA and PAAP remains highly individualized, with treatment decisions influenced by patient comorbidities, aneurysm morphology, anatomical considerations, technical feasibility, cost-effectiveness, and patient preferences. Current evidence suggests that OSR and endovascular repair should be viewed as complementary rather than competing modalities, with survival outcomes appearing broadly comparable despite significant baseline demographic differences in the populations selected for each approach. In particular, unfavorable anatomy for full endovascular treatment and in high-surgical-risk patients, the hybrid approach represents a viable option. Meticulous preoperative planning plays a pivotal role in obtaining successful outcomes and avoiding serious complications. While OSR may offer better long-term durability for younger, low-risk patients, endovascular repair may provide short-term benefits for higher-risk individuals. The choice between approaches should be made with careful consideration of all clinical and anatomical factors. Optimal outcomes are achieved in high-volume aortic centers with multidisciplinary expertise, where either open or endovascular, as well as the hybrid approach, can be used according to individual risk profiles.

## Figures and Tables

**Table 1:** PAAA—pararenal abdominal aortic aneurysm, PAAP—pararenal abdominal aortic pseudoaneurysm, JAAA—juxtarenal abdominal aortic aneurysm, TAAA—thoracoabdominal aortic aneurysm, SAAA—suprarenal abdominal aortic aneurysm, cAAA—complex abdominal aortic aneurysm, AAA—abdominal aortic aneurysm, IAAA—infrarenal abdominal aortic aneurysm, PAU—penetrating aortic ulcer, OSR—open surgical repair, EVAR—endovascular aortic repair, FEVAR—fenestrated endovascular aortic repair, BEVAR—branched endovascular aortic repair, ChEVAR—chimney endovascular aortic repair, F-BEVAR—fenestrated-branched endovascular aortic repair, F/BEVAR—fenestrated or branched endovascular aortic repair, N/A—not available.

Authors	Year	Country	Number of Patients	Age (Years)	Type of Aneurysm	Treatment Strategy	Mean Follow-Up (Months)	30-Day Mortality	Survival
van Lammeren GW et al. [20]	2017	Netherlands	214	69.8 ± 7.1	PAAA	OSR	20	3.4%	5 years—74.2%
Reyes et al. [21]	2016	Spain	34	74	PAAA after OSR for AAA (22 PAAP)	Endovascular 17 FEVAR 11 BEVAR 1 FEVAR + BEVAR 4 ChEVAR 1 “sandwich”	23.2 ± 16.6	3%	1 year—93.9% 2 years—90.9%
Mirza et al. [22]	2019	USA	243	75 ± 8	TAAA 147 PAAA 96	F-BEVAR	38 ± 15	2.5%	N/A
Werlin et al. [23]	2019	USA	162	73 ± 8	TAAA 73 PAAA 89	Endovascular	28	3.08%	N/A
Scali et al. [24]	2015	USA	37	67 ± 10	TAAA 24 PAAA 6 Pseudoaneurysm 3 Dissection 2 PAU 2	F-BEVAR	10.3	19%	1 year—70% ± 8% 4 years—67% ± 8%
Tshomba et al. [25]	2022	Italy	119	71.7 ± 6.8	JAAA 37 PAAA 57 SAAA 18 Type IV TAAA 7	OSR	76	1.7%	3 years-83.2% ± 3.4% 5 years-73.1% ± 4.1% 8 years-54.7% ± 6.2%
Zlatanovic et al. [5]	2023	Italy Serbia Netherlands Finland	834	73 ± 6.6 EVAR 69.5 ± 7.1 OSR	JAAA 483 PAAA 351	Endovascular 234 OSR 600	87	4.1% endovascular 5.5% OSR	38.6% EVAR 42.1% OSR
Wang et al. [26]	2022	China	10	54.5 ± 14.2	TAAA PAAA	Endovascular	30	0%	90%
Gallitto et al. [27]	2024	Italy Germany Sweden Greece Portugal France	197	75 ± 8	JAAA 64 PAAA 95	Endovascular	19 ± 5	11%	3 years 58%
Ferrer et al. [28]	2024	Italy	183	64.5 ± 5.7	JAAA 44 PAAA 33 TAAA 106	F/BEVAR	65.7 ± 39.6	2.2%	1 year 94.0% 5 years 85.1% 10 years 72.2%
Yeung et al. [29]	2024	Netherlands Italy Spain	42	76 ± 6	IAAA 6 JAAA 33 PAAA 3	Endovascular	3	2.4%	N/A
Sultan et al. [30]	2024	Ireland	99	74.7 ± 9.3 EVAR 73.2 ± 7.3 OSR	PAAA	EVAR 63 OSR 36	42.17 ± 32.38 EVAR 50.96 ± 38.8 OSR	0% EVAR 2.78% OSR	N/A
Branzan et al. [31]	2021	Germany	17	70 ± 9	PAAA TAAA	Endovascular	14.4	0%	N/A
Escobar et al. [4]	2022	USA	208	71 ± 8	TAAA 163 PAAA 45	Hybrid	21	14.4%	1 year 77 ± 3% 5 years 61 ± 5%
Zhang et al. [32]	2024	China	15	63.4 ± 10.7	TAAA 9 PAAA 6	Endovascular	31.4	0%	93%
Ribeiro et al. [33]	2025	Portugal	293	N/A	cAAA	EVAR 35.2% F/BEVAR 32.7% OSR 32.1%	N/A	N/A	N/A
D’Oria et al. [34]	2021	Sweden	202	72 ± 8	PAAA TAAA	F-BEVAR	32.8	2%	3 years 75.2%
Yazar et al. [35]	2025	Netherlands	23	72.3 ± 7.2	PAAA	iBEVAR	15	8.3%	78.3%
Biggs et al. [36]	2022	USA	32	74 ± 9	PAAA 10 TAAA 22	F-BEVAR	24 ± 22	6%	1 year 70% ± 8%
Wang et al. [37]	2025	China	115	69.1	JAAA PAAA	Endovascular	1	0.86%	N/A
Rastogi et al. [38]	2022	USA	1486	N/A	JAAA 575 PAAA 911	FEVAR	N/A	2.4%	3 years 89.5%
Gallitto et al. [39]	2021	Italy	221	N/A	TAAA 110 J/PAAA 111	F/BEVAR	27	4%	1 year 89% 3 years 75% 5 years 65%
Shibata et al. [40]	2024	Japan	121	75.6 ± 7.6	PAAA 62 TAAA 59	Endovascular	24.2	5.8%	3 years 83.3% PAAA 54.1% TAAA
Bisdas et al. [41]	2024	Greece Cyprus	21	71	PAAA	Endovascular	14 ± 7.7	0%	95%
Pitoulias et al. [42]	2022	Greece Germany Italy USA Austria	267	N/A	PAAA	ChEVAR	25.5 ± 13.3	1.9%	3 years 81.0%
van der Riet et al. [43]	2021	Netherlands Germany	194	72.2 ± 8.0	PAAA	FEVAR	24.6	3%	3 years 77%
Piazza et al. [44]	2023	Italy	116	73 ± 8	TAAA PAAA/P	Endovascular	3	N/A	94.8%
Farber et al. [45]	2025	USA	102	N/A	TAAA 59 PAAA 43	Endovascular	12	0%	1 year 94.1%
Latz et al. [46]	2020	USA	443	N/A	JAAA 253 PAAA 59 SAAA 100 Type IV 31	EVAR 23.7% OSR 76.3%	N/A	30.5% EVAR 23.8% OSR 32.5%	N/A
Fenelli et al. [47]	2024	Italy Germany	41	71±10	J/PAAA 8 TAAA 33	F-BEVAR	21±16	0%	1 year 90% 2 years 84%
Le Houerou et al. [48]	2023	France Spain	42	75.1 ± 10	TAAA PAAA	Endovascular	24.7	4.5%	2 years 73%
D’Oria et al. [49]	2022	Sweden Italy Denmark	222	71.6	PAAA TAAA	F-BEVAR	N/A	N/A	5 years 61.6%
Xodo et al. [50]	2025	Italy	5	71 ± 9	JAAA PAAA	F/BEVAR	12.4 ± 3.6	0%	N/A
Nguyen et al. [51]	2024	USA	100	73.7 ± 7.0	TAAA 42 PAAA 58	Endovascular	24	2%	2 years 87%

## Data Availability

No new data were created or analyzed in this study.
